# Supplementary material for: Recurrent E. coli Urinary Tract Infections in Nursing Homes: Insight in Sequence Types and Antibiotic Resistance Patterns
Source: Antibiotics (Basel). 2022 Nov 16;11(11):1638. doi: 10.3390/antibiotics11111638 (PMC9686610; doi:10.3390/antibiotics11111638)
Supplement: Supplementary file 1 [file antibiotics-11-01638-s001.zip › Table S2 supplementary v2 16112022.pdf]

Supplementary material

Table S2: Antibiotic resistance rates based on *E. coli* isolates from PROGRESS study

	E. coli episode from single UTI episode		*E. coli* episode from recurrent UTI group (recurrence)		All *E. coli* episode from both single and recurrent episode (overall)	
	**N**	**Resistance %**	N	**Resistance**	**N**	**Resistance %**
**Amoxicillin**	80	**37.5**	67	**43.3**	148	**39.9**
**Amoxicillin-clavulanic acid**	83	**36.1**	65	**40.0**	148	**37.8**
**Cefotaxime**	81	**4.9**	66	**4.5**	147	**4.8**
**Cefoxitin**	79	**21.5**	64	**17.2**	143	**19.6**
**Ceftazidime**	81	**2.5**	67	**4.5**	148	**3.4**
**Ceftriaxone**	81	**4.9**	67	**4.5**	148	**4.7**
**Ciprofloxacin**	81	**9.9**	67	**10.4**	148	**10.1**
**Cotrimoxazol**	81	**13.6**	67	**9.0**	148	**11.5**
**Fosfomycin**	81	**1.2**	67	**1.5**	148	**1.4**
**Gentamicin**	81	**3.7**	67	**0.0**	148	**2.0**
**Imipenem**	81	**0.0**	67	**0.0**	148	**0.0**
**Meropenem**	81	**0.0**	67	**0.0**	148	**0.0**
**Nitrofurantonin**	81	**6.2**	67	**1.5**	148	**4.1**
**Trimethoprim**	81	**19.8**	67	**9.0**	148	**14.9**
**Tobramycin**	79	**2.5**	64	**0.0**	143	**1.4**
